# ExerG: adapting an exergame training solution to the needs of older adults using focus group and expert interviews

**DOI:** 10.1186/s12984-022-01063-x

**Published:** 2022-08-16

**Authors:** Nathalie Ringgenberg, Sarah Mildner, Marcia Hapig, Sarah Hermann, Katharina Kruszewski, Anna Lisa Martin-Niedecken, Katja Rogers, Alexandra Schättin, Frank Behrendt, Sonja Böckler, Stefan Schmidlin, Roman Jurt, Stephan Niedecken, Christian Brenneis, Leo H. Bonati, Corina Schuster-Amft, Barbara Seebacher

**Affiliations:** 1 Department of Sport, Exercise and Health, University of Basel, Basel, Switzerland; 2 Department of Health Sciences, IMC University of Applied Sciences Krems, Krems, Austria; 3 Research Center on Vascular Aging and Stroke, VASCage GmbH, Innsbruck, Austria; 4 Research Department, Reha Rheinfelden, Rheinfelden, Switzerland; 5 Department of Design, Subject Area Game Design, Zurich University of the Arts, Zurich, Switzerland; 6 Sphery Ltd, Zurich, Switzerland; 7 Stratford School of Interaction Design and Business, University of Waterloo, Waterloo, ON Canada; 8 Department of Neurology, Clinic for Rehabilitation Münster, Münster, Austria; 9 Karl Landsteiner Institute of Interdisciplinary Rehabilitation Research, Münster, Austria; 10 Department of Neurology, University Hospital Basel, Basel, Switzerland; 11 Department of Clinical Research, University of Basel, Basel, Switzerland; 12 Institute of Rehabilitation and Performance Technology, Bern University of Applied Sciences, Burgdorf, Switzerland; 13 Department of Rehabilitation Research, Clinic for Rehabilitation Münster, Münster, Austria; 14 Clinical Department of Neurology, Medical University of Innsbruck, Innsbruck, Austria

**Keywords:** Focus groups, Exergaming, Virtual reality, Older adults, Geriatrics, Rehabilitation, User-centered design, Exercise rehabilitation

## Abstract

**Background:**

Exergames are playful technology-based exercise programs. They train physical and cognitive functions to preserve independence in older adults (OAs) with disabilities in daily activities and may reduce their risk of falling. This study gathered in-depth knowledge and understanding of three different user groups’ experiences in and relevant needs, worries, preferences, and expectations of technology-based training, to develop an exergame training device for OAs.

**Methods:**

We conducted a qualitative study using semi-structured focus group interviews of primary (OAs in geriatric or neurological rehabilitation) and secondary (health professionals) end users, as well as expert interviews of tertiary end users (health insurance experts or similar), exploring user perspectives on adjusting an existing exergame to OAs’ needs. Voice-recorded interviews were transcribed by researchers and analyzed using thematic analysis (TA) following an inductive, data-driven, iterative approach.

**Results:**

We interviewed 24 primary, 18 secondary, and 9 tertiary end users at two rehabilitation centers in Austria and Switzerland. Our TA approach identified five to six themes per user group. Themes in the primary end user group reflected aspects of safety, training goals, individuality, game environment, social interactions, and physical and technical overload. Themes in the secondary end user group comprised facets of meaningfulness, distraction through the game environment, safety, gamification elements, the availability and accessibility of the exergame. Tertiary end users’ themes addressed aspects of financial reimbursement, suitable target populations, professional training for the handling of exergame devices, training goals, and concerns about the use of exergames in geriatric rehabilitation.

**Conclusions:**

In conclusion, an exergame for OAs must be safe, motivating and fully adaptable to the target group while promoting the return to or preservation of autonomy and independence in daily life. Our findings contribute to developing hard- and software extensions for the ExerG training device. Further research is needed to expand the validity of our findings to larger populations.

**Supplementary Information:**

The online version contains supplementary material available at 10.1186/s12984-022-01063-x.

## Background

Approximately one third of older adults (OAs) aged 65 and over fall at least once a year. The fall incidence increases with age and frailty, which stresses the need for targeted interventions [1]. Fall-related costs are increasing annually worldwide, placing a burden on affected individuals, families, and the health care systems [2]. Several factors underlie an increased risk of falls in OAs, with the most significant being functional limitations, balance and gait impairments [3]. These frequently result from an age-related loss of muscle mass, bone density, and muscle strength [4]. Aside from physical limitations, cognitive impairment increases the fall risk [5, 6] and represents a major cause of fall-related fractures, particularly in combination with osteoporosis [3]. Injuries or hospitalizations may increase the fear of another fall in individuals, followed by avoidance behavior and progressive immobility, which in turn accelerates the loss of muscle mass and promotes further falls [4].

Physiotherapy interventions are recommended to reduce the risk of falls and should include strength and walking training, as well as function-based exercises [7]. In addition, the results of a recent meta-analysis support the implementation of cognitive exercises in physical activity programs [8]. Playful technology-based exercise programs, known as exergames, can boost performance motivation and adherence [9]. The word exergame is a combination of the terms “exercise” or “exertion” and “video game” and refers to a training approach with which video games are played through body movements [10]. Furthermore, exergames may create an ecologically valid training environment as they resemble activities of daily living such as walking, climbing stairs, shopping, and preparing meals [11]. Other authors have suggested that older individuals’ fall risk decreases following training using innovative technologies [12]. Through gaming environments, older individuals are distracted from their actual exercise performance and effort, which is associated with higher levels of motivation [12]. Generally, OAs prefer exercises relevant to their everyday lives, supporting the use of training activities of daily living within an exergame program [13]. The rapid advancement of digital technologies and limited experience in the digital domain can lead to a barrier to utilizing innovative technologies in older individuals [14]. To enable OAs to safely use exergames, and avoid overwhelming them which might cause rejection of the experience, games must be adapted in an age-appropriate manner [15].

So far, there are few providers who specialize in exergames for OAs and even fewer who provide specific exergame solutions for geriatric rehabilitation, among them being Dividat Senso (Dividat AG, CH) and Silverfit Virtual Cycling (Silverfit B.V., NL). In this study, we aimed to gain in-depth knowledge and understanding of primary (older adults, PEU), secondary (health professionals, SEU), and tertiary end users’ (health insurance experts or similar, TEU) previous experiences in technology-based training, as well as their needs, worries, preferences, and expectations, to develop an exergame training solution for OAs. A deeper understanding of end users’ experiences, needs, worries, preferences, and expectations gained in this study will help to develop the concept for hard- and software modification of an exergame training solution (ExerG). The future ExerG is expected to provide diverse training opportunities in the physical and cognitive domains. These training opportunities could help reducing the risk of falls. The research question guiding this study was: what are the end users’ experiences, needs, worries, preferences, and expectations towards an exergame training solution for OAs?

## Methods

### Study design

**Fig. 1:**
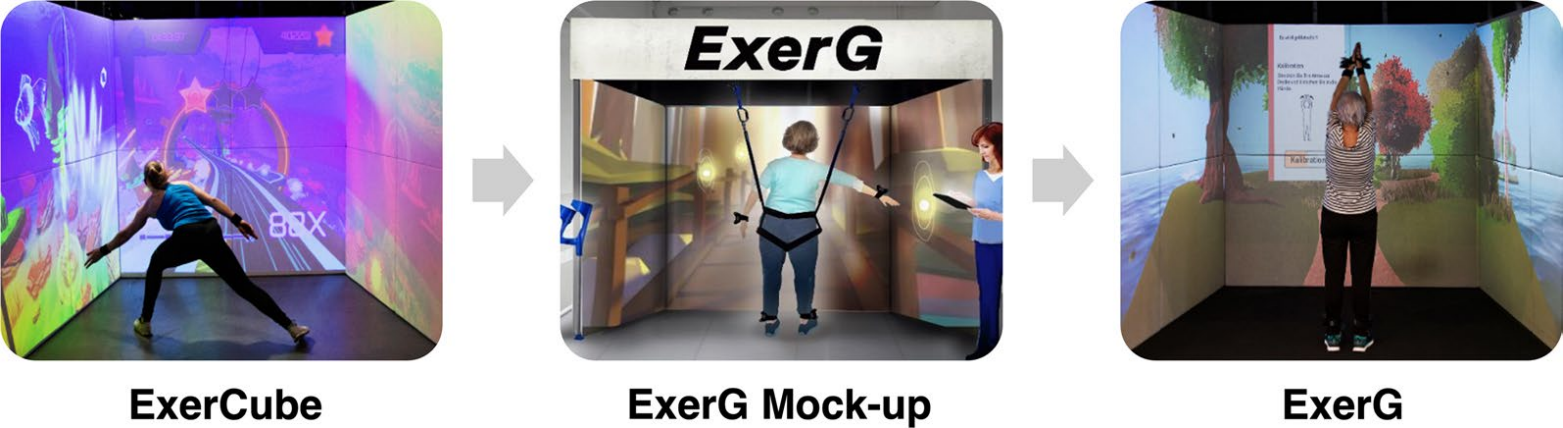
Adaptation process of an exergame training device (ExerCube) for older adults (ExerG) ©Sphery, CH

This study used a qualitative approach consisting of semi-structured interviews to gain in-depth knowledge and understanding of PEUs’, SEUs’, and TEUs’ experiences, needs, worries, preferences, and expectations regarding technology-based exercise training, specifically exergaming. Findings will be used to extend the ExerCube fitness exergame [16, 17] to the ExerG to target the needs of OAs. Figure 1 shows the initial exergame (ExerCube), first illustrations (ExerG mock-up) and the first prototype of the target exergame device (ExerG).

**Fig. 2:**
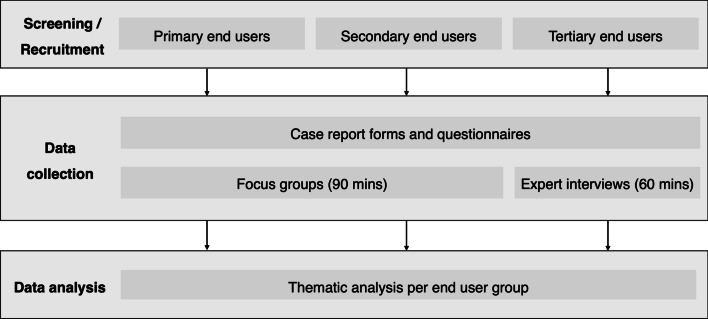
Overview of study procedures

Information reported in this study follows the qualitative research review guidelines (RATS, see Additional file 1) and Standards for Reporting Qualitative Research (SRQR, see Additional file 2) [18, 19]. Figure 2 presents the study procedures.

The focus of this study was the subjective experience of the target groups. Therefore, an inductive qualitative approach was chosen, to holistically capture participants’ thoughts [20]. Thus, OAs with all their complexities were at the center of the investigation. To capture this complexity, an analytic interpretive orientation was chosen that attempts to stay close to the participants' voices in the collected data, specifically the “hermeneutics of empathy” according to Braun and Clarke [20]. The aim of this approach is to understand the participants’ reality or views on topics and make sense of it [20].

In the PEU and SEU groups, the interviews were conducted as a focus group to encourage discussion among the participants. The group setting was intended to encourage participants to add to each other's thought processes in order to elicit as much as possible about the topic [21]. The group size (aiming for 20 PEUs, 16 SEUs, 4 TEUs) was set to be small enough to allow each participant to have their say and large enough to achieve a diversity of opinion [22]. Participants were allocated to focus groups on a pragmatic approach based on the time of their consent for participation. TEUs were specifically selected and interviewed individually using expert interviews [23].

### Sampling

Using convenience sampling, the rehabilitation centers (Reha Rheinfelden, RHF, Switzerland; Clinic for Rehabilitation Münster, RZM, Austria) recruited PEUs, SEUs, and TEUs.

The PEUs were recruited from the pool of in-patients with neurological, neuro-orthopedic and geriatric diseases of the respective rehabilitation centers who are prone to falls. Eligibility criteria for study participation in the PEU group were being ≥ 65 years old (a), cognitive status allowing them to understand the study procedure/content and informed consent (b), able to walk with or without a supportive device for 10 m, or if wheelchair-dependent, being able to sit in a wheelchair without arm and back rests (c). PEUs were excluded if they presented with a joint contracture (shoulder, knee, hip) (d), psychiatric disease (e), terminal illness with a life expectancy of less than 12 months (f) or intense pain during movement (> 5/10 points on the Visual Analogue Scale [24]) (g).

The SEUs consisted of physiotherapists, occupational therapists, sports scientists, and psychologists working at the rehabilitation centers. The TEUs were health insurance executive experts working for national (AUT, CH) or international governmental or non-governmental health associations.

### Recruitment

The PEUs and SEUs were recruited at the rehabilitation centers RZM and RHF. Daily patient entry lists were screened for potential candidates, who were approached by a project member to inform them about the study in oral and written form and the voluntary participation opportunity. Upon providing initial consent, interviewees were screened based on the eligibility criteria. If all criteria were met, an appointment for a focus group interview was scheduled. Participants did not miss therapy sessions from participating in the focus groups. For the recruitment of SEUs, emails were sent to all therapists of the respective rehabilitation centers asking if they were interested in participating. After initial agreement, tentative dates were offered to agree on a group interview date. TEUs were informed about the project by email. Contact details were selected from personal contacts or previous collaborations. After agreement to participate, a date for an individual online interview was scheduled. All participants provided their written informed consent.

### Focus group / interview setting and guide

Three to six PEUs / SEUs participated in each focus group on location, while TEUs were interviewed individually online. Focus group interviews were conducted in a quiet conference room with all interviewees sitting around a table. A visual interview presentation was given to support the discussion. Short breaks were included when necessary. One researcher acted as a moderator and led through the interview and discussion, while another researcher was responsible for technical support and taking field notes.

A semi-structured interview guide using open-ended questions was prepared for each end user group and applied identically in both rehabilitation facilities [25, 26]. The dimensions and questions of the guideline were developed based on different models and existing questionnaires mainly coming from the field of Human–Computer-Interaction, such as the Game Flow Model [27] the Dual Flow Model [28] and the Physical Activity Enjoyment Scale [29].The guideline went through several iterations and was used in other previous exergame projects [17, 30] before it was adapted for purpose of the focus groups and expert interviews presented in this paper. Questions were designed to stimulate focus group discussion in the focus group, allowing the moderator the flexibility to explore certain topics with open and follow-up questions (see Additional file 3). The openness of the questions was necessary to allow for diverse perspectives in the field of inquiry whilst still providing the necessary interview structure [23]. To ensure a basic level of mutual understanding, the topic and some technical terms were defined at the beginning. All SEUs already had experience with the use of exergames. The TEUs reported to have utilized exergames themselves, observed patients training with exergames or to be at least familiar with the goals and types of exergames. Introductory questions about individual experiences with games, movement, and technology were asked, followed by questions about technology and usability of exergames, data protection, exercise training, and social aspects. The ExerG training device concept was presented to all user groups using a prototype video and illustrations to gain a broad understanding of exergames. The presentation was adjusted to potential auditory and visual impairments of OAs and therefore pre-tested with six PEUs and four SEUs to refine the wording of the questions and better comply with the proposed duration of 90 min. For the TEUs, a specific semi-structured interview guide was used.

### Data collection and management

Demographic and professional data such as age, profession, work experience, previous knowledge of or experience with technology-assisted training were collected by screening medical records of the PEUs, direct questioning, or an on-site (RZM) or online (RHF) questionnaire. The Mini Mental State Examination (MMSE) [31] was used for assessing cognitive function, with a cut-score of < 24 indicating cognitive impairment [32], and the Barthel Index (BI) [33] and Extended Barthel Index (EBI) [34] were employed for evaluating daily functioning.

With participants’ consent, all interviews were digitally recorded on two redundant digital voice recording devices to avoid loss of data. Recorded interview data were transcribed verbatim and anonymized according to the extended transcription rules of Dresing and Pehl [35] by a researcher of the respective rehabilitation centers or a professional transcription service (Transkripto, Rotterdam, Netherlands), depending on the time resources of the study teams. For transferring the recorded interview files to Transkripto, the company signed a non-disclosure agreement, and the data were transferred through a Secure Sockets Layer computer network to guarantee secure communication. To verify the appropriateness of the transcripts, a researcher from the respective rehabilitation centers conducted a peer-check of several randomly selected transcript passages.

### Qualitative data analysis: coding and theme development

After confirmation of the transcripts’ accuracy by a peer, qualitative data were analyzed inductively using Thematic Analysis (TA). This approach was chosen because it provides a great deal of flexibility in analysis and can be applied to different ontological and epistemological positions. TA provides a set of basic core techniques and skills that are also used in other types of qualitative analysis [36]. TA identifies and organizes themes that appear important to the analyst for answering a research question [37]. MAXQDA (VERBI GmbH, GER) and f4 (dr. dresing & pehl GmbH, GER) software were used for thematic coding.

We chose an inductive, data-driven coding approach for our TA. After familiarization with the data sets, codes were derived a posteriori through examination of the generated data [38]. By coding bottom-up from data, a realist ontology and constructivist epistemology were used to generate an understanding of interviewees’ subjective experiences and motivations [20]. Ontological realism claims that there is a reality independent from human minds whereas constructivism maintains that people create new knowledge through interaction and sharing of their ideas and experiences [39]. Using an iterative process, two independent coders analyzed the transcripts separately per center for PEUs, SEUs, and TEUs.

**Fig. 3:**
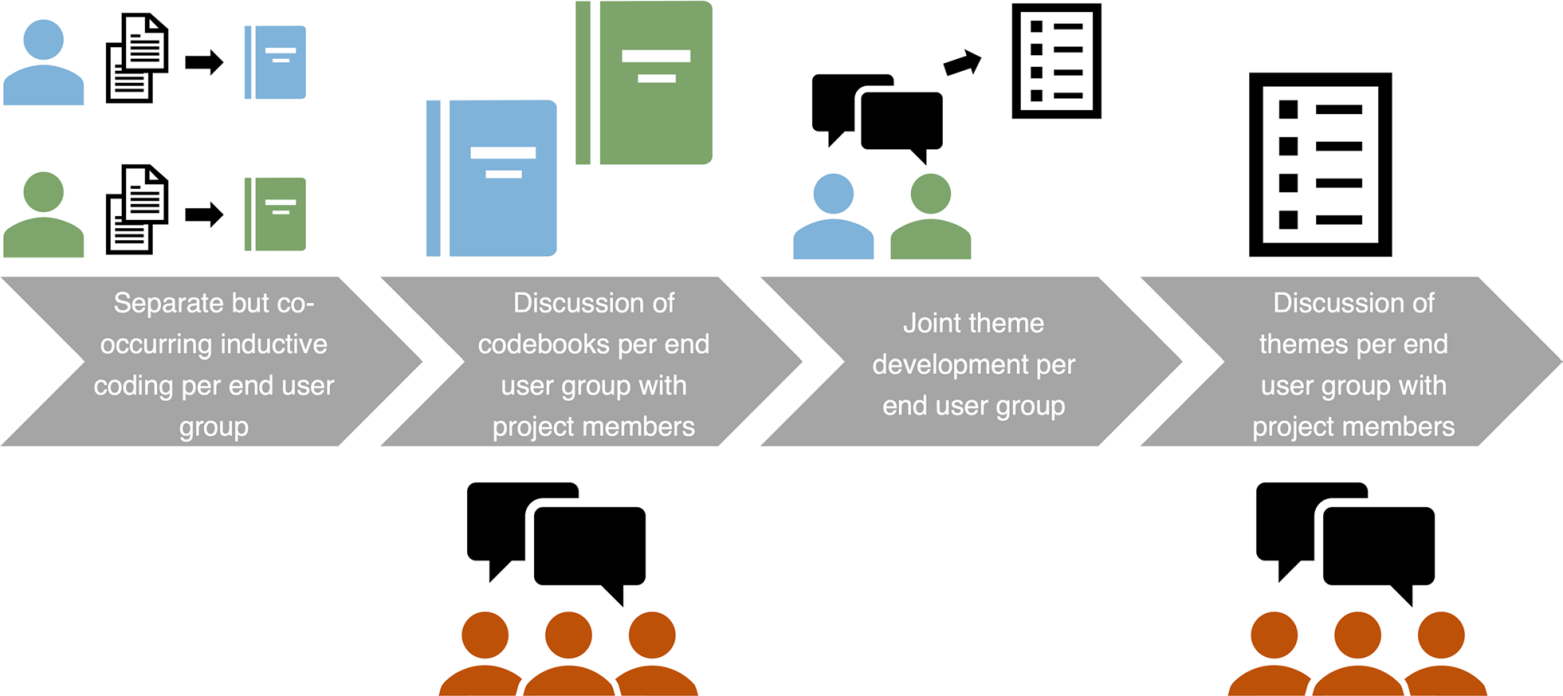
Thematic analysis process in this study

Separately developed codebooks were then discussed among researchers from both centers to establish similar standards for theme development (see Fig. 3). Subsequently, themes were developed together using various rounds of discussions among coders. Themes were revised and refined several times by two junior researchers who were supported by five experienced researchers. All themes should be understood interpretively because the statements are speculative and not definitive. This process was repeated for SEUs and TEUs.

### Statistical analysis

Statistical analyses were performed using IBM SPSS software, version 27.0. (Armonk, NY). Descriptive statistics were used for the demographic, professional, and disease-specific variables. Due to the small sample size, no inferential statistics were performed. Continuous data were checked for a normal distribution using the Shapiro Wilk Test, Q-Q plots, and histograms. Raw count (absolute and relative frequencies, N (%) was presented for count (walking aids) and nominal data (gender, profession, education level). Medians (minimum–maximum; 25th and 75th percentiles) were reported for ordinal data (MMSE, BI and EBI) and mean (standard deviation, i.e., SD) for continuous data (age, years of education and professional experience, and number of falls).

## Results

### Participants’ characteristics

**Fig. 4:**
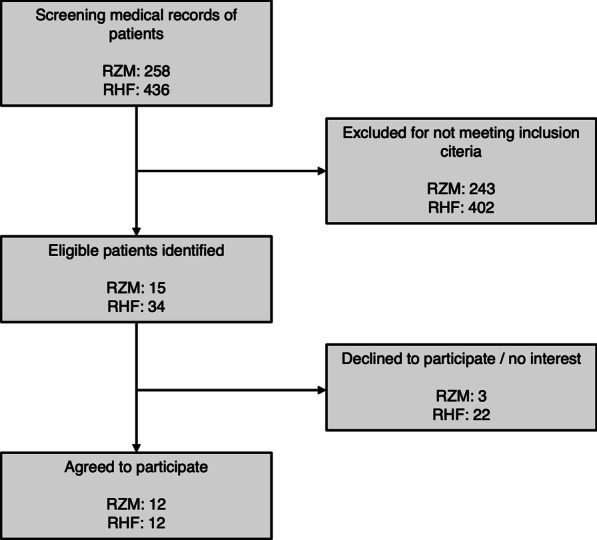
Flow chart of primary end user study participants recruited in Reha Rheinfelden (RHF) and Clinic for Rehabilitation Münster (RZM)

The study was conducted from August to December 2021. In total, 24 PEUs, 18 SEUs, and nine TEUs were included. Figure 4 shows the flow chart for the PEU group.

Focus groups with PEUs each included six patients at RZM and three patients at RHF, respectively. Mean age of PEUs was 75.7 (65–82) years. Focus groups in SEUs each included five therapists or psychologists at RZM and four therapists at RHF, respectively. SEUs’ mean age was 35.3 (25-52) years. Participant characteristics of PEUs, SEUs, and TEUs are presented in Table 2.

**Table 1:** Participant characteristics of primary, secondary, and tertiary end users

Primary end users (n = 24)	
Age in years (Mean (standard deviation; minimum–maximum))	75.7 (5.8; 65–82)
Gender: males / females (Frequency (percentage))	15 (62.5) / 9 (37.5)
Walking aid* (Frequency (percentage))	11 (45.8)
Mini Mental State Examination (Median (25th, 75th percentiles; minimum–maximum))	29 (27, 30; 24–30)
Barthel Index (Clinic for Rehabilitation Münster) (Median (25th, 75th percentiles; minimum–maximum))	100 (83, 100; 70–100)
Extended Barthel Index (Reha Rheinfelden) (Median (25th, 75th percentiles; minimum–maximum))	59 (56, 62; 45–63)
Fall prevalence over the last six months (Frequency (percentage))	No fall 9 (37.5) Up to five times 5 (20.8) More than five times 1 (4.2)
Diagnoses (Frequency (percentage))	Ischemic or hemorrhagic cerebrovascular insult 8 (33.3) Parkinson’s disease 6 (25) Multiple sclerosis 2 (8.3) Tick-borne encephalitis 1 (4.2) Lumbar spinal stenosis with hip flexor paresis 1 (4.2) Structural epilepsy 1 (4.2) Polytrauma 1 (4.2) Spinal disc herniation 1 (4.2) Polyarthrosis, osteochondrosis with vertigo 2 (8.3) Multimorbidity 1 (4.2)

Secondary end users (n = 18)	
Age in years (Mean (standard deviation; minimum–maximum))	35.3 (7.5; 25–52)
Gender: males / females (Frequency (percentage))	8 (44.4) / 10 (55.6)
Profession (Frequency (percentage))	Physiotherapist 10 (55.6) Occupational therapist 2 (11.1) Sports scientist 4 (22.2) Psychologist 2 (11.1)
Work experience in years (Mean (standard deviation; minimum–maximum))	9.0 (6.2; 0.5–20)
Education level (Frequency (percentage))	Bachelor´s degree 4 (22.2) Master´s degree or equivalent 9 (50.0) Doctoral degree 3 (16.7) Others 2 (11.1)

Tertiary end users (n = 9)	
Age in years (Mean (standard deviation; minimum–maximum))	49.7 (9.4; 31–59)
Gender: males / females (Frequency (percentage))	6 (66.7) / 3 (33.3)
Work experience in years (Mean (standard deviation; minimum–maximum))	26.7 (10.7; 4–38)
Education level (Frequency (percentage))	Bachelor´s degree 1 (11.1%) Master´s degree or equivalent 3 (33.3) Doctoral degree 4 (44.4) Others 1 (11.1)

^*^Use of a wheelchair or walking aid such as rollator or walking stick

### Primary end users’ perspectives on exergames

We identified six themes through TA in our PEU focus group data (Table 3).

**Table 2:** Themes resulting from the thematic analysis of primary end users’ data

Primary end users	
1	The game environment distracts OAs from the physical and mental effort during training
2	Social relationships are of great importance and should be included in an exergame either through physical presence of therapists or direct interaction with therapists or co-players
3	Due to the age- or illness-related limitation of their personal agency, OAs desire safety during the training
4	OAs worry about feeling physically as well as technically overwhelmed. A lack of experience and concerns regarding the exposure to computer game addiction additionally leads to hesitancy regarding technology use
5	Narratively realistic training that focuses on ADL is desired to manage daily living as independently as possible
6	A wide variety of individual customization options are desired to increase motivation through successful exergame experiences

*PEU*  primary end user, *OAs*  older adults, *ADL*  activities of daily living

These themes highlight the complexity of PEUs’ experiences, needs, worries, preferences, and expectations for an exergame training solution for OAs. In the following, we present the themes with key example quotes. For all themes identified, more quotes from the interviews can be retrieved from the appendix (Additional file 4).

1. **The game environment distracts OAs from the physical and mental effort during training.**

PEUs expressed that the game environment could reduce boredom and monotony during training. This distraction would allow OAs to exercise in a more relaxed manner. A bicycle path or forest trail as a digital environment could motivate them to exercise:

P08: “Yes, I could imagine that you take a bit of the boredom out of the training program at home, when you work out on the home trainer using training equipment. And at the same time, you see a route that you like and that interests you: a bike path or a forest trail where you can do biking as well. Of course, that would be much more motivating. I could continue the path next time and drive across Austria, for example.” (RZM_PEU2, paragraph 82)

2. **Social relationships are of great importance and should be included in an exergame either through physical presence of therapists or direct interaction with therapists or co-players.**

It became clear that PEUs very much appreciate social interaction during their training. Personal care was considered important by OAs and must not be replaced by devices:

P05: “Of course it is interesting, all these devices and suggestions. What I would miss is the human being in it, the personal attention. Or being cared for, or whatever.” (RZM_PEU1, paragraph 493)

Social interaction can occur with co-players, as well as with therapists. Interviewees described that training with co-players could create cohesion and ambition. Therapists’ influence during training also seemed to matter to participants. A strong and trusting relationship with the therapist was considered essential. In their opinion, the therapist is responsible for guiding exercise performance and interpersonal aspects (e.g., motivating or showing empathy).

P17: “[I need someone] who knows me inside out and instructs: ‘You have to speed up’/ (.) well, I cannot hang out there in a cool way/ (.) no, you have got a tough guy at the back there who can give you a little push! Right? And/ (.) but the praise comes at exactly the same time: ‘Hey, great, you showed a good commitment!’ And the therapist knows you.” (RHF_PEU4, paragraph 698)

3. **Due to the age- or illness-related limitation of their personal agency, OAs desire safety during the training.**

This theme reflects patients’ physical limitations due to their age or illness. To avoid injury, PEUs desired to be safe during training. With optimum safety precautions, such as the presence of a therapist or using handrails, they expect they can actively participate in the game.

P01: “Yes, it [safety] is very important, because I am not allowed to fall anymore.” (RZM_PEU1, paragraph 316)

The ExerG training device will be equipped with a safety harness to prevent falling. According to one participant, surfaces that are touched as part of the game tasks should be soft to minimize the risk of injury. This would allow participants to feel safe enough to independently perform the exergame tasks:

*P06: “Yes, to put on the harness, this safety thing first, and maybe give instructions: ‘Nothing will happen to you and now it will take a quarter of an hour’. And when you touch something, it has foam rubber or something like that (.). Then no one would have to stand by me (.) during the [game].” (RHF_PEU1, paragraph 465).*

According to some participants, the feeling of being secured would increase one’s self-confidence to move outside the usual comfort zone.

4. **OAs worry about feeling physically as well as technically overwhelmed. A lack of experience and concerns regarding the exposure to computer game addiction additionally leads to hesitancy regarding technology use.**

PEUs do not consider themselves digital natives. They have little to no experience with technology-assisted therapy and often think critically about it. Participants were afraid of being overwhelmed, suggesting that a user-friendly interface adapted to them is of key importance. Despite the user-friendly interface, training with the ExerG should only occur after professional training of the OA by SEUs, illustrated by the following example:

*P05: “I think that is good, but it does not seem so easy and for me it would be very difficult to take part in it. I am interested, but I find it very demanding. At least considering my present condition. Very difficult. I would almost be a little overwhelmed with certain things.” (RHF_PEU1, paragraph 375)*

Factors for OAs' disinterest in exergames ranged from general disinterest to insufficient knowledge about the use of digital devices. Another factor contributing to the distrust of computers or exergames was the fear of game addiction, which was mentioned only by a few participants:

*P10: “An online card game, I cannot think of the name now (...). I played that passionately, almost addictively, and with a lot of effort I got out of the habit, because I realized how long I spend playing [online] and that is not good for me. I do not play other games at all/. I am not interested in that.” (RZM_PEU2, paragraph 37)*

5. **Narratively realistic training that focuses on activities of daily living (ADL) is desired to manage daily living as independently as possible.**

Independent coping with everyday life is a high priority for PEUs. The data showed that training tasks which are closely related to everyday life activities within an exergame intervention would present a way to increase motivation. A mock-up video of an exergame that can be used to simulate shopping at the supermarket was shown to the focus group participants as an instrumental activity of daily living. Patients’ responses demonstrated an increased interest in using an exergame if situations close to everyday life can be trained:

*P10: “It was very close to reality, so it was easy to empathize. [...] My first thought was that I could relate to it.” (RZM_PEU2, paragraph 238)*

Self-stated goals of the PEUs reflected the importance of ADL. Above all, the independent ability to walk was in the focus. Patients stated that an improvement visible in their ADL would motivate them to continue exercising. The following excerpt shows that the PEUs were aware of their ability to achieve their goals through regular training in therapy:

*P05: “There must be more stability (..) I am an insecure walker or (.) sometimes a little dizzy and so on and I am here to improve that with a therapist. Learning to walk properly again or and that is the main goal, that I walk again, independently (.) like before.” (RHF_PEU1, paragraph 220)*

In addition to the ability to walk, hobbies were also regarded as important. These are often no longer possible, due to physical limitations or the risk of falling.

6. **A wide variety of individual customization options are desired to increase motivation through successful exergame experiences.**

This theme reflects various customization ideas for the exergame suggested by the PEUs. The type of activity, but also the difficulty level should be adjustable according to the interviewees:

P08: “But I still see a big advantage with computer assisted video games, you can introduce practically any level or any performance standard. I can do penalty shootouts today against Christiano Ronaldo and this is facilitative, to make the game more exciting or increasingly more exciting. Or I can play tennis against Dominic Thiem. It is all conceivable.” (RZM_PEU2, paragraph 164).

With adaptive levels of difficulty, the interviewees expect they would be able to perceive improvements that they make themselves. Several participants mentioned this as a way to promote motivation and stimulate performance. Exergames should therefore enable the visualization of small improvements:

P15: “Then it becomes difficult to maintain one’s motivation. […] Yeah, if you do not see the progress.” (RHF_PEU4, paragraphs 561–563)

### Secondary end users’ perspectives on exergames

We identified six main themes through TA in our SEU focus group data (Table 4).

**Table 3:** Themes resulting from the thematic analysis of secondary end users’ data

Secondary end users	
1	From the SEUs’ perspectives, a functional and individualized meaningful game design is of great importance to OAs for increasing training motivation
2	The fall protection device in the ExerCube is expected to provide OAs with a feeling of safety that will allow them to train at their individual performance limits
3	Based on the therapists' experience, digital gamification in therapy leads to an increased motivation in OAs
4	The game environment provides an opportunity to distract OAs from their functional limitations and allows them to unconsciously move more freely
5	The availability and perceived time demands of an exergame may limit its usability
6	A lack of local accessibility to and availability of the exergame after discharge from a rehabilitation center and an unawareness of alternative, non-computerized training strategies may influence adherence negatively

*SEUs*  secondary end users, *OAs*  older adults

1. **From the SEUs’ perspectives, a functional and individualized meaningful game design is of great importance to OAs for increasing training motivation.**

SEUs expressed that training goals must be individually selected and specified very precisely, allowing OAs to find them meaningful to themselves and motivating them to exercise in rehabilitation:

*S02: “I also have the impression, [… that] if they simply train their balance specifically for THAT [ADL or sports activities], where they need it, they are also more motivated and simply (...) more involved in the therapy than just doing some exercises, where they do not know whether they need them in everyday life.” (RZM_SEU1, paragraph 21)*

Regarding the exergame, SEUs desired high quality and realistic graphics, sound, and story. They imagined natural game environments (e.g., a forest or city walk) being more attractive to OAs than a virtual fantasy environment. They suggested ‘useful’ and functional activities like picking mushrooms in the forest instead of touching points of light to achieve a high score in a racing game. In their opinion, training should be adapted to individual functional goals. Functional exercises which are meaningful and relevant for their everyday lives, should be provided for various sports activities or ADLs in the exergame. A supermarket visualization where OAs perform everyday movements (e.g., a type of squats) as training was particularly well received:

*S08: “Somehow transfer that [personal interests] into the game. Or then you picked things up, did squats or something like that/. […] And then you walk through the store because you want to take something out of the bottom shelf. Well, I think that calls for such a system.” (RHF_SEU2, paragraphs 1082-1084)*

Examples of other conceivable activities for exergames reported in the focus groups were cooking, cleaning, washing clothes, gardening, or hiking in nature.

2. **The fall protection device in the ExerCube is expected to provide OAs with a feeling of safety that will allow them to train at their individual performance limits.**

SEUs regarded a fall protection device as essential for OAs’ safety feeling during therapy. Consequently, they would have greater courage or even train at their individual performance limits, which was likely to promote training progress:

*S03: “Being able to try out everything in a SAFE setting that the patient would no longer have DARED to do: walking backwards, walking forwards, walking sideways, changing quickly, and then throwing himself in. That was where I think a lot of TRUST can be re-established, if the [performance] limit is secured.” (RZM_SEU1, paragraph 52)*

Interviewees also stated that falls cannot be fully prevented and might be provoked in a therapy setting to conduct an effective training. In their opinion, training at the individual performance limit is only possible if an OA is not completely secured and fixed, so that movements can be carried out independently, naturally, and in the full range of motion:

*S02: “And I still find it difficult, especially when it comes to balance training [...]. I mean, the Dividat is great, it has a railing around it where patients can hold on to. But if they hold on then we will not have the effect we want. […]. To find such a/ such a good balance between safety and effectiveness. So, effective balance, I would find that kind of difficult.” (RHF_SEU2, paragraphs 507-511)*

However, all of the SEUs agreed that training at an individual performance limit is highly important for a successful therapy. A training session should be doable but still challenging.

3. **Based on the therapists' experience, digital gamification in therapy leads to an increased motivation in OAs.**

SEUs considered it highly beneficial, making the therapy playful. Digital gamification e.g., using exergames and playful elements were suggested for enhancing OA’s motivation. Interviewees reported a high intrinsic motivation in OAs:

*S01: “Our feedback from MANY people is just like, 'What, the therapy is already finished yet?' And now, no, they want to keep playing because they are going to crack the high score again, and the motivation is just there.” (RZM_SEU1, paragraph 76)*

According to SEUs, the playful nature of exergames allow to make monotone, simple and repetitive movements more exciting for OAs. Another benefit was that people may be distracted from their continuous self-observation by the game environment. SEUs assumed that a higher motivation and engagement during therapy with exergames were associated with an external focus of attention:

*S03: “The advantage is that tasks are solved playfully, adopting an external focus [of attention]. And by doing so, you almost forget the time or the effort. That is also the effect that you otherwise (.) had with a ball back then, if you do something with the ball, you forget the time and effort, yes. You do a lot more with it than when you say: ‘now you run five laps in the hall. That's a huge difference, yes.” (RHF_SEU1, paragraph 178)*

However, SEUs were convinced that OAs will lose interest very quickly if they would not notice success. It was therefore very important to implement partial goals into the exergame and thereby create feelings of success.

4. **The game environment provides an opportunity to distract OAs from their functional limitations and allows them to unconsciously move more freely.**

SEUs speculated that patients may not focus on their own limitations (e.g., reduced range of motion, pain, fatigue, and mental stress) but an engaging play with exergames. Consequently, they were more willing to give a little more effort:

*S01: “Well, for example, he is someone who really likes to walk on his heels. And now, when you bring the bees [in an exergame] to the front/ (.) like, then you notice: Hopp, he is in trouble right away. But at least he does it. And sometimes during the exercises, no matter how I tell him: ‘Hey, now do not shift the weight forward with your forefoot’/ (.) difficult! (laughs) [...] But here in this game, that is what happens.” (RHF_SEU2, paragraphs 102-104)*

Therapists mentioned that patients may forget a rehabilitation setting by playing exergames, making them remember other places and activities. Overall, this distraction was perceived as a positive element of exergames.

5. **The availability and perceived time demands of an exergame may limit its usability.**

SEUs wished for an easy-to-use exergame. It was particularly important to them that the harness can be applied easily and patients introduced to the game quickly, so that no therapy time was wasted. Updates, software or graphical issues or necessary adjustments prior to the training discouraged SEUs from introducing the exergame to their patients:

*S07: “For me it's also very important, or (smiles) almost a top priority, that the device works (collective laughter) properly. That may sound banal (S04 laughs), but it happens more often than you think […]. If, as a therapist, you somehow have no idea what the problem is, then it affects the patient, and they might not like to train as much next time. So, it just has to work (.) or to be solved quickly (common laughter). I see that as a bit of a barrier, that there are always technical errors, software things or maybe hardware issues.” (RHF_SEU1, paragraph 163)*

SEUs indicated that they depend on the proper functioning of an application or training device, since the total duration of therapy is about 30 min. Therefore, the functioning should be self-explanatory and guaranteed. Some SEUs preferred to use equipment they were already familiar with, as they often lacked the time to try different settings. It seemed that a well-functioning of technical devices was not guaranteed overall, as shown by the ironic laughter of all interviewees in a focus group:

*S10: “Well, I think it definitely adds value if you [...] use the device, I think that is very exciting. [However, currently it is] difficult setting everything up and adjust it individually to the patient but at the same time still be very easy to use [...] so that it almost always works at the push of a button […], that would of course be the (laughter from everyone) ultimate highlight for a therapist.” (RZM_SEU2, paragraph 393)*

6. **A lack of local accessibility to and availability of the exergame after discharge from a rehabilitation center and an unawareness of alternative, non-computerized training strategies may influence adherence negatively.**

Due to financial and spatial requirements of the exergame, SEUs worried that OAs would not continue their exercise routine after having left the rehabilitation institution because of difficulties accessing the exergame outside the rehabilitation setting (e.g., in physiotherapy):

*S08: “Yes, for the patients in inpatient settings it is always important that they can perhaps continue somewhere at home or in therapy. […] I mean, if you […] have equipment, then they always ask: ‘Ah, which private practice has it, too?’” (RHF_SEU2, paragraph 1142)*

Therapists argued that a dependency of an exergame could arise and therefore they suggested occasionally using the exergaming device as an add-on to conventional therapy:

*S02: “I think, in principle it is good as a SUPPLEMENT to therapy, I would definitely NOT REPLACE therapy with it, so that you only do the exergame […] The only danger I might see with something like that is that a certain dependency might occur.” (RZM_SEU1, paragraph 88)*

Therefore, to the SEUs it appeared important to provide OAs with exercises, which can be performed individually with little help of a professional or specific training device.

### Tertiary end users’ perspectives on exergames

Five main themes were identified through TA of our TEUs' focus group data (Table 1).

**Table 4:** Themes resulting from the thematic analysis of tertiary end users’ data

Tertiary end users	
1	From the TEUs’ perspectives, an evidence-based additional benefit is a prerequisite for financially supporting a research and development project, as well as for considering a financial reimbursement of exergame applications within rehabilitation
2	TEUs desire diverse training applications and settings of the ExerG in order to reach heterogeneous target populations with different impairments and at various rehabilitation stages
3	The main goal of an ExerG training should be OAs’ return to an autonomous everyday life
4	TEUs assume that OAs are reluctant to use exergames, due to a lack of experience in technology and therefore express themselves partially skeptical, critical and/or reserved about the use of the ExerG in rehabilitation settings
5	A professional training of the therapist and patient-oriented training support during exergaming are considered vital for therapeutic success

*TEUs*  tertiary end users, *OAs*  older adults

1. **From the TEUs’ perspectives, an evidence-based additional benefit is a prerequisite for financially supporting a research & development project, as well as for considering a financial reimbursement of exergame applications within rehabilitation.**

TEUs agreed on the importance of an evidence-based additional benefit through the ExerG training. This was one of the most frequently used concerns TEUs pointed out when asked about their opinion on reimbursement of the exergame training, as well as the future establishment of the finalized training device in rehabilitation and similar settings. They emphasized the necessity of manifest proofs of efficacy, as illustrated by the following quotation:

*T03: “If we talk about health insurances, we have the case, that they prefer to see evidence-based [proof of efficacy], that a real benefit is gained from using that device” (RZM_TEU3, paragraph 22)*

Generally, open-mindedness towards the future use of technology-based training interventions in the field of rehabilitation and prevention was indicated, associated with a tentative intention to support research and development projects financially:

*T01: „On the one hand we obviously have the commission to achieve the optimum for our clients, for your patient, who is the same person, to be able to support the optimal possibility for healing. The financial component, however, is still the first priority for us, where there has to be some kind of [reasoning] for it to become obvious: ‘Okay, that is financially still attractive’. Maybe you could therefore also state, yes, maybe that [the training with the exergame] could result in a reduction of the duration of being inpatient or something similar. It is not about inventing incentives in whatever way, but there should be a progress somehow/. There clearly has to be a progress, a considerable advantage derived from the training, which you could state. Therefore we need some information, but for, for the support of pilot projects we are PRINCIPALLY open.” (RHF_TEU1, paragraph 99)*

2. **TEUs desire diverse training applications and settings of the ExerG in order to reach heterogeneous target populations with different impairments and at various rehabilitation stages.**

TEUs showed interest in the innovative training device and expressed their open-mindedness by discussing various training applications and settings. Adopting an economical perspective, they revealed creativity with respect to novel ideas about future training settings (e.g., in nursing homes). They regarded it very important to integrate possibilities for adapting the level of difficulty to the patient:

*T03: “And for whom would that be suitable? If there is a certain case like patient A for an individual application and then there is another individual application for patient B and in the end, it is only poorly and rarely in use. I think in that case it makes sense to say: Okay, you can apply it reasonably throughout one’s therapeutic treatment at various, I would call them levels of progression or stages of progression.” (RHF_TEU3, paragraph 273)*

Although there was a common idea about the use of technology-based training interventions like the ExerG, one TEU was concerned about a broad application of the innovative device:

*T02: “I think it is certainly EXCITING. (.) As I said before, you specifically have to select and choose, what people are we talking about if we talk about patients (.) right? Which indicators are given? Therefore, you cannot speak for ALL, right? But you have to select very specifically what patients are in need and could get something out of it.” (RHF_TEU2, paragraph 32)*

3. **The main goal of an ExerG training should be OAs’ return to an autonomous everyday life.**

From the perspective of TEUs, the goal and purpose of a rehabilitation stay is for OAs to regain autonomy and independence. Patients’ return to an autonomous daily life would be a reduction in costs to the health care system. For this reason, TEUs suggested a game design similar to the patient's daily life to train these skills. The following passage illustrates the benefits of using an everyday environment (in this case, a supermarket):

*T01: “Well, what I like about it, is that it is not based on, not machinery controlled, but for real you have to stand and walk, bend, like natural movements, needed in everyday life. And that is also presented in this way by those videos or these games. And that you regain your capacity of how to do the groceries.” (RHF_TEU1, paragraph 191)*

It was speculated that people, who are reluctant to use technology-based training devices probably would approach games with an everyday life background more easily and motivated:

*T02: “It is pleasurable, that the world presented is not a VIRTUAL one [...] but being presented as a day-to-day-world, right? For example being at (the river?) or in a supermarket, I think it is basically a good concept (.) which may also simplify the access for people who are not that much into gaming, in my opinion” (RHF_TEU2, paragraph 92)*

4. **TEUs assume that OAs are reluctant to use exergames, due to a lack of experience in technology and therefore express themselves partially skeptical, critical, and/or reserved about the use of the ExerG in rehabilitation settings.**

Various TEUs were concerned about the actual usability of the developed software and hardware system in relation to the target population. Their concerns as to overburdening OAs are illustrated by the following quotation:

*T05: “The first videos were appropriate for young people, not for the older generation. This is not suitable for an old person, also rather dissuasive, if there are so many things which impact on you while standing in a virtual room and there are just too many different impressions. An older person is not capable of processing, and may feel more threatened rather than comfortable, without getting a real benefit out of it.” (RZM_TEU5, paragraph 36)*

It was pointed out that the purpose of the ExerG training should be clarified in advance and the patient informed before the training session starts.

5. **A professional training of the therapist and patient-oriented training support during exergaming are considered vital for therapeutic success.**

TEUs agreed on the significant importance of personal support and company throughout the whole ExerG training session, provided by a trained specialist or therapist. According to the TEUs, the therapeutic relationship must not suffer, deteriorate or disrupt due to establishing technology-based interventions in rehabilitation settings. To them it was essential that patients are provided with a feeling of security and social interaction during their rehabilitation stay. The following passage illustrates this fundamental need:

*T01: “In the future, the personal contact within a therapeutic process must not be replaced completely, [the new development should be combined] with being trained or treated by another human being, and I see it as an add-on [treatment] instead of a replacement.” (RZM_TEU1, paragraph 27)*

It was further pointed out that the supervision by a trained specialist or therapist was a crucial prerequisite for a successful implementation of the training:

*T03: “I am concerned that, let us say, okay, for example you play a game, where you perform squats (.) I would call it, make somebody jump. It does not matter, you perform whatever action. But then it is also important that you check that: ‘Like okay (.) is that task completed correctly?’ As you may get into the flow of gaming, not being able to follow the tasks appropriately and you make mistakes by performing your exercises, and in the end, you worsen more than you improve.” (RHF_TEU3, paragraph 64)*

Additionally, a correct introduction through a trained specialist or therapist was declared as an important component of the therapy success. In summary, the TEUs mostly were open-minded towards the idea of the project but conveyed at the same time some concerns regarding a training focus on everyday life and required proofs of efficacy.

## Discussion

In our study, we aimed to identify the end users’ experiences, needs, worries, preferences, and expectations of an exergame training solution for OAs. We begin the discussion of our findings by reflecting on our methodology. We identified our themes in a reflexive process. This means our results are coupled with our prior knowledge, thoughts, and views on our research topic. Different researchers performed the coding of transcripts from both rehabilitation centers individually. Interestingly, we used different codes but came to agreement in terms of developing themes, which in our opinion presents the data from both rehabilitation centers in meaning. The different codes showed how our backgrounds differently influenced the reflexive coding process. The creation of the themes was influenced by the coders' different backgrounds in the fields of sport and exercise science, physiotherapy, and clinical rehabilitation. Additionally, researchers in the fields of game design and human–computer interaction were involved, bringing their perspectives into the theme development.

In all end user groups’ data, there was one theme relating to OAs’ wish to preserve or return to autonomous and independent daily living. This shows its high perceived importance in all end user groups, and matches the literature which widely describes this as a key factor of quality of life in OAs [40]. Based on the data, the ExerG could be an appropriate approach to promote autonomy and independence, not least because the ExerG tasks can be designed to be similar to ADL, where OAs respond to stimuli by planning their actions. Additionally, we identified in our data that OAs prefer activities that are meaningful to them and their lives. A supermarket setting was particularly positively perceived in all end user groups. As quality of life and activities of daily living are coupled in many ways, we suggest choosing a game design that is set close to the everyday life of OAs and includes motor activities they need on a daily basis. This may promote a faster and more sustained progression in rehabilitation of OAs (e.g., shorter stay in a rehabilitation clinic). As our TEU groups emphasized a perceivable functional benefit as necessary, using exergames in rehabilitation appears beneficial. However, systematic reviews and meta-analyses have only shown small positive impacts of exergames on health-related quality of life in OAs [41, 42]. Due to large heterogeneity in study procedures and small sample sizes, more studies are necessary to investigate the effects of exergames in OAs, especially those undergoing rehabilitation.

Safety was perceived as highly important to the PEUs and SEUs. SEUs viewed the fall protection device as essential to create a safe training environment. A safety-effectiveness trade-off was put forward by SEUs who emphasized the importance of securing patients as little as possible but also as much as necessary; this enables patients to encounter fall-risk situations and learn from them. Therapists stressed that an exergaming session should be demanding. This aligns with the literature, as exercise programs for fall prevention that challenge balance and are of higher exposure have shown larger effects in fall prevention [7]. PEUs highlighted that the presence of a therapist and professional support during exergaming would be relevant to enhance both their perception of safety and their motivation. Prior work suggests incorporating either human-based or automated fall detection in exergames [43]; this is partly in agreement with our data; assistance from a human being was perceived as essential in our interviews.

PEUs and SEUs expressed their desire for individualized adaptation to increase motivation through a sense of achievement. Individually adjustable game elements may serve to expand the application to suit diverse populations and settings, as requested by TEUs. Some studies have highlighted the need for pursuing personalized goals and performance levels in exergames [44, 45], which is in line with our study where adjustments in difficulty, distance, and speed were desired. The automatic adaptation of the exergame to their current performance level seemed particularly relevant to our end users. This means that the game difficulty would increase with a good performance and decrease with a poor performance.

The playful nature of exergames was considered positive in our PEU and SEU groups, which agrees with studies that have found that playing games during therapy correlates with a distraction from pain, effort, and physical or mental exhaustion [12, 46]. According to a variety of studies, exergames’ playful nature evokes positive emotions in OAs, which are in turn likely to enhance motivation and free movements [47–51]. Results from both our and other studies suggest that the game narrative should be drawn from enjoyment such as sporting activities, or familiar activities such as shopping [52]. As OAs have different interests and preferences, it seems useful to include simple games with easily identifiable goals (e.g., collecting or identifying objects). Interestingly, in our study, patients described playful sports activities as a positive design angle for an exergame, even if they were not able to perform them in real life anymore.

Social interaction was considered important by the PEUs: they wished for either the presence of a therapist or multiplayer options for co-playing with peers during their therapy routine. The relevance of social interaction during exergaming, including a trusting relationship with the therapist if they are involved, has widely been discussed in the literature [53, 54]. These studies have suggested incorporating high-score boards to promote a feeling of connectedness with peers. In addition, our results revealed that the motivation to play could remain high when the scores of similarly skilled competitors were displayed on a high-score board.

Our results indicated that due to a lack of knowledge and experience, OAs showed a negative attitude and were hesitant about using a new technology. Other studies have found further barriers to the use of technology such as age-related vision and hearing loss and impairment in fine motor skills [55]. All these factors were emphasized in our focus groups and expert interviews as well, and, in accordance with the literature, it was suggested that simple instructions and explanations should be provided for OAs [56]. In addition, TEUs advised professional training for therapists prior to instructing patients to facilitate exergaming effectiveness.

SEUs emphasized that an exergame for OAs should simply work using a user-friendly interface and without redundant adjustments prior to starting the game. Similarly, studies have shown that a stepwise introduction to and reduced complexity of information improves the willingness to use exergames in OAs [45, 57].

### Strengths and limitations

This study interviewed three user groups that are relevant for the development of an exergame. PEUs, SEUs, and TEUs were involved to obtain a broad picture of their experiences, needs, worries, preferences, and expectations of an exergame in general. We expanded the perspectives even further through data collection in two rehabilitation centers in different countries. Some challenges were associated with interviewing the different population groups as they brought different requirements and knowledge about technology-based rehabilitation into the discussions. The interdisciplinary consortium on the project including researchers within different areas of expertise was a strength of this study. Through discussions with experts from different fields it was possible to expand the view on topics.

TA is highly dependent on a researcher’s position and their interpretation of content [58]. It is therefore not guaranteed that other researchers would find the same results in our data. It means our understanding of the data and our shaping of the themes is informed by our prior knowledge of sports and movement science, physiotherapy and by game design and human–computer interaction, and clinical rehabilitation expertise. However, we aimed to achieve transparent data analysis by explaining our reflexive process carefully and comprehensively. The researchers who primarily analyzed the data were not experts in TA. Advice was provided however from experienced researchers within the research team.

Another limitation is the theoretical nature of the questions being asked to the end users, as users were not able to directly interact with the ExerCube or ExerG prototype but rather were presented with a video demonstration before answering the interview questions. We attempted however to minimize this limitation by verbal explanations and presenting videos of the previous ExerCube and the mock-up videos of the new ExerG. All participants were thus provided with the same information and invited to imagine themselves training in the ExerG and freely express their thoughts.

An unequal gender distribution may have been a further limitation of our study, with the sample of both PEUs and TEUs being nearly 2/3 male. For the SEUs, there were 10 females and 8 males. Furthermore, there were equal numbers of females and males among PEUs in the focus groups at RZM but not in RHF. Similar codes and the same themes for SEUs were generated for both centers despite the gender imbalance and independent coding. For the TEUs, it cannot be completely ruled out that slightly different results could have been obtained in more gender-balanced groups.

Another limitation in our study could have been a bias of social desirability. The group dynamic might have influenced individuals’ opinion and what was expressed during discussions. As PEUs were rather not familiar with exergames, their imagination and shared knowledge may have been influenced by the discussions.

Given the small sample size and gender imbalance, we may not have achieved saturation, which is another limitation of this study. Further studies are needed to validate our results.

## Conclusions

This study provides an interpretive understanding of three end users groups’ experiences, needs, worries, preferences, and expectations of an exergame device, as a basis for adapting an existing exergame, the ExerCube, to the needs of OA.

Based on the results of our study, the following six main factors will help to develop the ExerG: incorporate a safety system and a safety harness for the patients (a); include different performance levels in the design of the games (b); create realistic games to encourage a return to autonomous everyday life (c); offer high-score boards to maintain a high level of motivation (d); enable quick and easy harness application (e); include partial goals in the games and provide a lot of feedback on the achieved performance in order to maintain a high level of motivation (f). In conclusion, an exergame for OAs must be safe, motivating and fully adaptable to the target group while promoting the return to or preservation of autonomy and independence in daily life. These findings contribute to developing hard- and software extensions for the ExerG training device. Further research is needed to extend the validity of our findings to larger populations of OAs.

## Supplementary Information

**Additional file 1.** Qualitative Research Review Guidelines (RATS) checklist, Completed RATS checklist with page number information of the main manuscript text.

**Additional file 2.** Standards for reporting qualitative research (SRQR) checklist, Completed SRQR checklist with page number information of the main manuscript text.

**Additional file 3.** ExerG—Semi-structured interview guide, Interview guide with procedures and prepared questions for the semi-structured interviews with primary, secondary, and tertiary end users.

**Additional file 4.** Detailed list of end users’ verbatim quotations, Verbatim quotations, and supplementary quotes from the interviews for primary, secondary, and tertiary end users.

## Data Availability

The datasets generated (transcripts, case report forms) and/or analyzed during the current study are not publicly available due to privacy reasons of participants but are available in an anonymized form from the corresponding author on reasonable request.

## Abbreviations

ADL: Activity of daily living
Ltd: Limited
OA: Older adult
PEU: Primary end user
RHF: Reha Rheinfelden
RZM: Clinic for Rehabilitation Münster
SEU: Secondary end user
TEU: Tertiary end user
TA: Thematic analysis
