# Supplementary material for: ExerG: adapting an exergame training solution to the needs of older adults using focus group and expert interviews
Source: J Neuroeng Rehabil. 2022 Aug 16;19:89. doi: 10.1186/s12984-022-01063-x (PMC9382774; doi:10.1186/s12984-022-01063-x)
Supplement: Supplementary file 1 — Additional file 1. Qualitative Research Review Guidelines (RATS) checklist, Completed RATS checklist with page number information of the main manuscript text. [file 12984_2022_1063_MOESM1_ESM.pdf]

Additional file 1: Qualitative Research Review Guidelines (RATS) checklist

The RATS guidelines modified for BioMed Central are copyright Jocalyn Clark, BMJ. They can be found in Clark JP: **How to peer review a qualitative manuscript**. In *Peer Review Health Sciences*. Second edition. Edited by Godlee F, Jefferson T. London: BMJ Books; 2003:219-235

Item	Guide questions/description	Reported (Page #)
**R – Relevance of study design**		
Is the research question interesting?<br>Is research question relevant to clinical practice, public health, or policy?	Research question explicitly stated<br>Research question justified and linked to the existing knowledge base (empirical research, theory, policy)	6<br>5-6
**A – Appropriateness of qualitative method**		
Is qualitative methodology the best approach for the study aims?<br>• *Interviews:* experience, perceptions, behavior, practice, process<br>• *Focus groups:* group dynamics, convenience, non-sensitive topics<br>• *Ethnography:* culture, organizational behavior,<br>• interaction<br>• *Textual analysis:* documents, art, representations, conversations	Study design described and justified e.g., why was a particular method (i.e., interviews) chosen?	6-8, Figure 2
**T – Transparency of procedures**		
Sampling<br>Are the participants selected the most appropriate to provide access to the type of knowledge sought by the study?<br>Is the sampling strategy appropriate?	Criteria for selecting the study sample justified and explained<br>• *theoretical:* based on preconceived or emergent theory<br>• *purposive:* diversity of opinion<br>• *volunteer:* feasibility, hard-to-reach groups	7-8
Recruitment<br>Was recruitment conducted using appropriate methods?<br>Is the sampling strategy appropriate?<br>Could there be selection bias?	Details of how recruitment was conducted and by whom<br>Details of who chose not to participate and why	8-9<br>8, Figure 4

Data collection<br>Was collection of data systematic and comprehensive?	Method (s) outlined and examples given (e.g., interview questions)	6-11, Additional file 3, Figure 3
Are characteristics of study group and setting clear?	Study group and setting dearly described	9, Table 1
Why and when was data collection stopped, and is this reasonable?	End of data collection justified and described	7-8
Role of researchers<br>Is the researcher(s) appropriate?	Do the researchers occupy dual roles (clinician and researcher)?	29-30, 32-33, 35, 36
How might they bias (good and bad) the conduct of the study and results?	Are the ethics of this discussed? Do the researcher(s) critically examine their own influence on the formulation of the research question, data collection, and interpretation?	32-33
Ethics<br>Was informed consent sought and granted?	Informed consent process explicitly and dearly detailed	8-9, 35
Were participants'' anonymity and confidentiality ensured?	Anonymity and confidentiality discussed	10
Was approval from an appropriate ethics committee received?	Ethics approval cited	35
**S – Soundness of interpretive approach**		
Analysis<br>Is the type of analysis appropriate for the type of study?<br>• *Thematic:* exploratory, descriptive, hypothesis generating<br>• *Framework:* e.g. policy<br>• *Constant comparison/grounded theory:* theory generating, analytical	Analytic approach described in depth and justified<br>Indicators of quality:<br>Description of how themes were developed from the data (inductive or deductive)<br>Evidence of alternative explanations being sought	6-7
Are the interpretations dearly presented and adequately supported by the evidence?<br>Are quotes used and are these appropriate and effective?	Analysis and presentation of negative or deviant cases<br>Description of the basis on which quotes were chosen<br>Semi-quantification when appropriate<br>Illumination of context and/or meaning, richly detailed	10-11, Figure 3
Was trustworthiness/reliability of the data and interpretations checked?	Method of reliability check described and justified e.g., was an audit trail, triangulation, or member checking employed?<br>Did an independent analyst review data and contest themes? How were disagreements resolved?	10-11

Discussion and presentation<br><br>Are findings sufficiently grounded in a theoretical or conceptual framework?<br><br>Is adequate account taken of previous knowledge and how the findings add?<br><br>Are the limitations thoughtfully considered?<br><br>Is the manuscript well written and accessible?	Findings presented with reference to existing theoretical and empirical literature, and how they contribute<br><br>Strengths and limitations explicitly described and discussed<br><br>Evidence following guidelines (format, word count)<br><br>Detail of methods or additional quotes, contained in appendix	29-33<br><br>32-33<br><br>7, Additional file 1, Additional file 2<br><br>Additional file 4
**Are red flags present?**<br><br>These are common features of ill-conceived or poorly executed qualitative studies, are a cause for concern, and must be viewed critically<br><br>They might be fatal flaws, or they may result from lack of detail or clarity	Grounded theory; not a simple content analysis but a complex, sociological, theory generating approach<br><br>*Jargon:* descriptions that are trite or jargon filled should be viewed skeptically<br><br>*Over interpretation:* interpretation must be grounded in "accounts" and semi-quantified if possible or appropriate<br><br>*Seems anecdotal, self-evident:* may be a superficial analysis, not rooted in conceptual framework or linked to previous knowledge, and lacking depth<br><br>*Consent process thinly discussed:* may not have met ethics requirements<br><br>*Doctor-researcher:* consider the ethical implications for patients and the bias in data collection and interpretation	

Once you have completed this checklist, please save a copy and upload it as part of your submission. When requested to do so as part of the upload process, please select the file type: *Checklist*. You will NOT be able to proceed with submission unless the checklist has been uploaded. Please DO NOT include this checklist as part of the main manuscript document. It must be uploaded as a separate file.
