# Supplementary material for: ExerG: adapting an exergame training solution to the needs of older adults using focus group and expert interviews
Source: J Neuroeng Rehabil. 2022 Aug 16;19:89. doi: 10.1186/s12984-022-01063-x (PMC9382774; doi:10.1186/s12984-022-01063-x)
Supplement: Supplementary file 2 — Additional file 2. Standards for reporting qualitative research (SRQR) checklist, Completed SRQR checklist with page number information of the main manuscript text. [file 12984_2022_1063_MOESM2_ESM.pdf]

Additional file 2: Standards for reporting qualitative research (SRQR) checklist

O'Brien BC, Harris IB, Beckman TJ, Reed DA, Cook DA. **Standards for reporting qualitative research: a synthesis of recommendations.** *Academic Medicine*, 2014;89(9):1245-1251. DOI: 10.1097/ACM.0000000000000388

http://www.equator-network.org/reporting-guidelines/srqr/

Item	Guide questions/description	Reported (Page #)
**Title and abstract**		
Title	Concise description of the nature and topic of the study Identifying the study as qualitative or indicating the approach (e.g., ethnography, grounded theory) or data collection methods (e.g., interview, focus group) is recommended	1
Abstract	Summary of key elements of the study using the abstract format of the intended publication; typically includes background, purpose, methods, results, and conclusions	3-4
**Introduction**		
Problem formulation	Description and significance of the problem/phenomenon studied; review of relevant theory and empirical work; problem statement	4-5
Purpose or research question	Purpose of the study and specific objectives or questions	6
**Methods**		
Qualitative approach and research paradigm	Qualitative approach (e.g., ethnography, grounded theory, case study, phenomenology, narrative research) and guiding theory if appropriate; identifying the research paradigm (e.g., postpositivist, constructivist/ interpretivist) is also recommended; rationale**	7
Researcher characteristics and reflexivity	Researchers' characteristics that may influence the research, including personal attributes, qualifications/experience, relationship with participants, assumptions, and/or presuppositions; potential or actual interaction between researchers' characteristics and the research questions, approach, methods, results, and/or transferability	29, 30, 33
Context	Setting/site and salient contextual factors; rationale**	9
Sampling strategy	How and why research participants, documents, or events were selected; criteria for deciding when no further sampling was necessary (e.g., sampling saturation); rationale**	7, 8, 9, Figure 4

Ethical issues pertaining to human subjects	Documentation of approval by an appropriate ethics review board and participant consent, or explanation for lack thereof; other confidentiality and data security issues	8, 9, 35
Data collection methods	Types of data collected; details of data collection procedures including (as appropriate) start and stop dates of data collection and analysis, iterative process, triangulation of sources/methods, and modification of procedures in response to evolving study findings; rationale**	10, 11, Figure 2
Data collection instruments and technologies	Description of instruments (e.g., interview guides, questionnaires) and devices (e.g., audio recorders) used for data collection; if/how the instrument(s) changed over the course of the study	9, 10, Additional file 3
Units of study	Number and relevant characteristics of participants, documents, or events included in the study; level of participation (could be reported in results)	8, Table 1
Data processing	Methods for processing data prior to and during analysis, including transcription, data entry, data management and security, verification of data integrity, data coding, and anonymization/de-identification of excerpts	10, 11
Data analysis	Process by which inferences, themes, etc., were identified and developed, including the researchers involved in data analysis; usually references a specific paradigm or approach; rationale**	10, 11, Figure 3
Techniques to enhance trustworthiness	Techniques to enhance trustworthiness and credibility of data analysis (e.g., member checking, audit trail, triangulation); rationale**	10, 11
**Results/findings**		
Synthesis and interpretation	Main findings (e.g., interpretations, inferences, and themes); might include development of a theory or model, or integration with prior research or theory	12-29, Table 2-4
Links to empirical data	Evidence (e.g., quotes, field notes, text excerpts, photographs) to substantiate analytic findings	12-29, Additional file 4
**Discussion**		
Integration with prior work, implications, transferability, and contribution(s) to the field	Short summary of main findings; explanation of how findings and conclusions connect to, support, elaborate on, or challenge conclusions of earlier scholarship; discussion of scope of application/generalizability; identification of unique contribution(s) to scholarship in a discipline or field	29-33
Limitations	Trustworthiness and limitations of findings	32-33
**Other**		
Conflicts of interest	Potential sources of influence or perceived influence on study conduct and conclusions; how these were managed	35
Funding	Sources of funding and other support; role of funders in data collection, interpretation, and reporting	36

*The authors created the SRQR by searching the literature to identify guidelines, reporting standards, and critical appraisal criteria for qualitative research; reviewing the reference lists of retrieved sources; and contacting experts to gain feedback. The SRQR aims to improve the transparency of all aspects of qualitative research by providing clear standards for reporting qualitative research.

**The rationale should briefly discuss the justification for choosing that theory, approach, method, or technique rather than other options available, the assumptions and limitations implicit in those choices, and how those choices influence study conclusions and transferability. As appropriate, the rationale for several items might be discussed together.

Once you have completed this checklist, please save a copy and upload it as part of your submission. When requested to do so as part of the upload process, please select the file type: *Checklist*. You will NOT be able to proceed with submission unless the checklist has been uploaded. Please DO NOT include this checklist as part of the main manuscript document. It must be uploaded as a separate file.
