# Supplementary material for: ExerG: adapting an exergame training solution to the needs of older adults using focus group and expert interviews
Source: J Neuroeng Rehabil. 2022 Aug 16;19:89. doi: 10.1186/s12984-022-01063-x (PMC9382774; doi:10.1186/s12984-022-01063-x)
Supplement: Supplementary file 3 — Additional file 3. ExerG—Semi-structured interview guide, Interview guide with procedures and prepared questions for the semi-structured interviews with primary, secondary, and tertiary end users. [file 12984_2022_1063_MOESM3_ESM.pdf]

Additional file 3: ExerG – Semi-structured interview guide

Primary end users

Welcome to the participant(s)

Introduction of moderator and research assistant

Outlining the aims of the focus-group discussion and providing an introduction to the topic

Obtaining informed consent for the voice recording

Introductory questions

- What does exercise mean to you in your everyday life?
- Please describe your most common forms of movement in detail.
- What is your attitude towards gaming in everyday life/in general?
- What is your attitude towards video games in everyday life/in general?
- Do you have any previous experiences with video games?
- Looking back, what new technologies were included in your therapy or training? Please describe these technologies and your experience(s).
- What experience with exergames do you already have? Please describe.

Questions about attitude and experience

- What is your attitude towards exergames? Please describe.
- How would it be for you to train your body and mind with the help of an exergame?

Questions about technology and usability

- What is your personal attitude towards new technologies? In general and specifically for therapy or training.
- What would prevent you from using new technologies (e.g., exergames) as a therapy or training device?
- What would assist you in your use of new technologies (e.g., exergames) as a therapy or training device?
- Let's think about the advantages and disadvantages of using new technologies (e.g., exergames) for therapy and training.

Questions about data security

- Which aspects do you find most important when it comes to protecting your data?
- Who do you think should have access to your therapy/training data?

Questions about training apects

- What are your main goals in your therapy or training? What physical and cognitive functions or everyday movements would you like to train/practice and improve?
- Do you think an exergame could train these functions? Why (not)?
- How do you assess aspects of safety during therapy or training?

Questions about willingness to train and motivation

- Think about your current therapy or training: how would you describe your motivation?

- Is there something that particularly motivates you when it comes to therapy or training?
- Could an exergame motivate you in therapy or training? Why (not)?
- Could anything be more motivating about an exergame than training with conventional methods?

Questions about social aspects

- What pros and cons can you think of when it comes to group training or therapy?
- Thinking about yourself – do the advantages of a group training outweigh the disadvantages or vice versa?
- How do you rate the importance of the relationship and communication with a therapist during therapy or training?

Presentation of the ExerG Prototype

- What do you think of the presented pictures, video and systems? What were your first thoughts?
- What (images, video and systems) did you like the most or sparked your interest straight away?
- What do you think about the usability of this system?
- What would speak for or against using such a system in therapy or training?
- What physical and cognitive functions should be trainable with this system?
- What movements could you envision training with this system?
- If you would train using this system, how long (per session) would you like to train and how often per week?
- Imagine the system adapting to your individual needs. Would that change your interest or motivation?

Questions about safety and ergonomics (hardware)

- How do you imagine a fall protection (harness) in the ExerCube?
- What could be the best way to put on a harness? Would you like to do this yourself?
- What role should your trainer/therapist play when putting on the harness (active, passive)?
- What would be the maximum reasonable effort (time) for you to put on a harness?
- Which of the presented security options seem to be the safest for you / seems to offer the most comfort / and degree of freedom for movements?
- How would you like a harness that not only secures but is also an active part of the game e.g., with buttons that are part of the game? What would you wish for in such a harness?

Final opinions, comments, and suggestions

- Is there anything else you think is important that we haven't discussed yet?
- Do you have any further remarks or suggestions?

Thanks for your participation and farewell

Secondary end users

Welcome to the participant(s)

Introduction of moderator and research assistant

Outlining the aims of the focus-group discussion and providing an introduction to the topic

Obtaining informed consent for the voice recording

Introductory questions

- How do you rate the importance of targeted training to improve your patient's balance and cognitive functions?
- Please describe what types of therapy or training you typically use for elderly patients who are at increased risk of falling. And why do you use them?

Questions about attitude and experience

- If you think about the therapy strategies, you use: Are there sometimes new technologies involved? Please describe.
- Do you already have experiences with exergames that you would like to share? (In what context were these experiences?)
- Please describe how you perceive the use of exergames in therapy and training.
- I would be interested in your personal attitude towards exergames - would you like to share this with us?
- What would it be like for you to use an exergame to train the physical and cognitive functions of patients?

Questions about the characteristics of their patients

- Which limitations of the patients do we have to expect (e.g., visual, auditory limitations) that must be considered in the development of an exergame?

Questions about technology and usability

- Now I would also be interested in your personal attitude towards new technologies – in general and for training or therapy.
- How do you perceive the attitude of your patients towards new technologies?
- Is there anything that prevents you as a therapist from using new technologies (e.g., exergames) as a therapy or training device?
- Is there anything that promotes your willingness as a therapist to use new technologies (e.g., exergames) as a therapy or training device?
- Let's think about the advantages and disadvantages of using new technologies (e.g., exergames) for therapy and training.
- What safety precautions do you think are important for new technologies (e.g., exergames) in the context of therapy and training?

Questions about data security

- Which aspects do you find most important when it comes to protecting personal data?
- Who do you think should have access to therapy/training data?
- Do you see any advantages in tracking patients' therapy/training data via mobile app and in adjusting the therapy/training content via mobile app?
- Would you use such an app? Please describe your thoughts to us.

Questions about training apects

- Which physical and cognitive functions or everyday movements would you like to train/improve with your patients?
- What would you expect from an exergame for a training? (What should such an exergame be able to train?)
- What criteria would an exergame have to meet in order for you to use it in your daily therapy or training routine?

- Are there therapy or training areas to be covered that are currently not yet or not reliably covered conventionally and that an exergame could cover?

Questions about willingness to train and motivation

- How do you motivate your patients during therapy or training?
- Please describe how you perceive the motivation of your patients? (Mostly extrinsically or intrinsically motivated?)
- Let's consider whether exergames can contribute to therapy or training motivation.
- Depending on the previous answers: Under what conditions could exergames be more motivating? To what extent do you see no advantage of exergames in terms of patient motivation for therapy?

Questions about social aspects

- What pros and cons can you think of when it comes to group training or therapy?
- How do you rate the importance of interacting with a patient during therapy or training?
- How do you interact with patients during therapy?

Presentation of the ExerG Prototype

- What do you think of the presented pictures, video and systems? What were your first thoughts?
- What (images, video and systems) did you like the most or sparked your interest straight away?
- What do you think about the usability of this system?
- What would speak for or against using such a system in therapy or training as a therapist?
- What could be problematic factors when using the device?
- Which motor and physical functions of your patients could you imagine to train with the system?
- Which cognitive functions of your patients could you imagine to train with the system?
- If you would train your patients in this system, how long (per session) would you train with them and how often per week?
- Imagine the system adapting to your patients' individual needs. Would that change your interest or motivation for using the device with patients?
- What do you think are important requirements for the design of an exergame for patients? Thinking about graphics, style, sound, story, etc.
- What else would you have to consider when designing the exergame (e.g. considering colors, visual stimuli, auditory stimuli)?

Questions about safety and ergonomics (hardware)

- How do you imagine a fall protection (harness) in the ExerCube?
- What is the best way to attach a harness to the patient?
- What role should the trainer play when putting on the harness (active, passive)?
- What is the maximum reasonable effort for putting on a harness? Should patients be able to do it by themselves?
- Which of the presented security options seems to be the best for your patients and why (safety, comfort, degree of freedom for movements)?
- How would you like a harness that not only secures but is also an active part of the game? e.g. with buttons that are part of the game. What would you wish for in such a harness?

Final opinions, comments, and suggestions

- How would you describe your general impression of the project?
- What aspects should be kept in mind during development?
- Do you have any further remarks or suggestions?

Thanks for your participation and farewell

Tertiary end users

Welcome to the participant(s)

Introduction of moderator and research assistant

Outlining the aims of the focus-group discussion and providing an introduction to the topic

Obtaining informed consent for the voice recording

Introductory questions

- Do you have experience with therapy and/or training systems that use new technologies for therapy or training with patients?
- Have you ever heard of the term "exergames"?
- Where do/did you already have contact with new technologies (e.g. exergames)?
- What is your personal attitude towards new technologies (e.g. exergames) - in general and for training or therapy?

Questions about training apects

- What do you think of training sessions in which patients' physical and cognitive functions are trained with the help of an exergame?
- Can you imagine exergames becoming an integral part of therapy and training processes?
- Are there therapy or training areas to be covered that are currently not yet or not reliably covered conventionally and that an exergame could cover?

Questions about technology and usability

- How important is it to you that new technologies are used in the therapy and training of patients?
- What criteria do new technologies (e.g., exergames) have to meet so that they would be (financially) supported or are of interest to you?
- What would prevent you from (financially) supporting an exergame for therapy or training?

Questions about data security

- Which aspects do you find most important when it comes to protecting personal data?
- Who do you think should have access to therapy/training data?
- Do you see any advantages in tracking patients' therapy/training data via mobile app and in adjusting the therapy/training content via mobile app?

Presentation of the ExerG Prototype

- What do you think of the presented pictures, videos and systems? What were your first thoughts?
- What (images, videos and systems) did you like the most or sparked your interest straight away?

- How would you describe your general impression of the project?
- Could you imagine supporting such a project or product?

Final opinions, comments, and suggestions

- What aspects should be kept in mind during development?
- Do you have any further remarks or suggestions?

Thanks for your participation and farewell
