# Supplementary material for: ExerG: adapting an exergame training solution to the needs of older adults using focus group and expert interviews
Source: J Neuroeng Rehabil. 2022 Aug 16;19:89. doi: 10.1186/s12984-022-01063-x (PMC9382774; doi:10.1186/s12984-022-01063-x)
Supplement: Supplementary file 4 — Additional file 4. Detailed list of end users’ verbatim quotations, Verbatim quotations, and supplementary quotes from the interviews for primary, secondary, and tertiary end users. [file 12984_2022_1063_MOESM4_ESM.pdf]

Additional file 4: Detailed list of end users' verbatim quotations

Primary end users

1. The game environment distracts older adults from the physical and mental effort during training.

P08: „Yes, I could imagine that you take a bit of the boredom out of the training program at home, when you work out on the home trainer using a training equipment. And at the same time, you see a route that you like and that [route] interests you: a bike path or a forest trail where you can do biking as well. Of course, that would be much more motivating. I could continue the path next time and drive across Austria, for example.“ (RZM_PEU2, paragraph 82)

Further examples from the interviews to theme 1 which are not cited in the main body of the manuscript

P11: „I rather think that that/ (.) that you could also be a bit more relaxed, perhaps with something like that. That you are not too tense.“ (RHF_PEU3, paragraph 1422)

P01: „Mountains range in front of me so that I can cycle for much longer. I hear the (inc.), I hear the kids as well as the room, there I always look at the clock and there I have the (inc.) in front of me and the whole landscape and you always hear something and the birds chirping in the evening. Therefore, time passes so quickly. I am distracted and I cycle along and the good air (…) and when it gets colder I move it [the home trainer] inside.” (RZM_PEU1, paragraph 127)

2. Social relationships are of great importance and should be included in an exergame either through physical presence of therapists or direct interaction with therapists or co-players.

P05: „Of course it is interesting, all these devices and suggestions. What I would miss is the human being in it, the personal attention. Or being cared for, or whatever." (RZM_PEU1, paragraph 493)

P17: „Who knows me inside and out and instructs: ‘You have to speed up’/ (.) well, I cannot hang out there in a cool way/ (.) no, you have got a tough guy at the back there who can give you a little push! Right? And/ (.) but the praise comes in exactly the same time: 'Hey, great, you showed good commitment!' And the therapist knows you / " (RHF_PEU4, paragraph 698)

Further examples from the interviews to theme 2 which are not cited in the main body of the manuscript

P06: „I think there is even some cohesion. I approach people. I have the first contact and do not ask three times: ‘What is your name?’ – NO. And then there is a proverb in Germany: Unity makes you STRONG. And I think that's good, because when I'm with people and I play with the balloon and practice, suddenly you are ONE, aren't you? (inc.) there and there and there and look what's your name, who is that, suddenly everyone has the ambition to shoot the balloon away, right? That is my opinion. Thus, the therapy in groups is better for me than one-to-one therapy. "(RZM_PEU1, paragraph 388)

P08: „Because I understand that under a social aspect, I see the advantage that you just have a lot more fun in a group than you do alone, it is not nearly as bland." (RZM_PEU2, paragraph 191)

P08: „For me, the therapist is like the teacher in school. The therapist knows, he is the expert, he knows what mistakes you make. He must take corrective action, intervene. And the better the relationship is with

the therapist, the easier it is and the more fun it is. And I think that fun is important, that humor is important in therapy." (RZM_PEU2, paragraph 218)

3. Due to the age- or illness-related limitation of their personal agency, older adults desire safety during the training.

P01: „Yes, it is very important, because I am not allowed to fall anymore.” (RZM_PEU1, paragraph 316)

P06: „Yes, to put on the harness, this safety thing first, and maybe give instructions: ‘Nothing will happen to you and now it will take a quarter of an hour’. And when you touch something, it has foam rubber or something like that (.). Then no one would have to stand by me (.) during the ride." (RHF_PEU1, paragraph 465).

Further examples from the interviews to theme 3 which are not cited in the main body of the manuscript

P04: „Safety is important. The close personal relationship with the trainer helps you to feel safe. He catches you before you are on the ground.” (RZM_PEU1, paragraph 339)

P07: „Yes there must be a security person present. Exactly. " (RHF_PEU1, paragraph 470)

P17: „Then maybe you also feel a little more confident to/ (.) to do more.“

I: „Yes. Ah, you think if you feel safe, then/”

P17: „Then maybe you also try a little higher/ (.) a track further.“

P15: „Yes.”

P16: „Hm (agrees)”

P17: „And do not give up immediately. " (RHF_PEU4, paragraphs 507-512).

4. Older adults worry about feeling physically as well as technically overwhelmed. A lack of experience and concerns regarding the exposure to computer game addiction additionally leads to hesitancy regarding technology use.

P05: „I think that is good, but it does not seem so easy and for me it would be very difficult to take part in it. I am interested, but I find it very demanding. At least considering my present condition. Very difficult. I would almost be a little overwhelmed with certain things." (RHF_PEU1, paragraph 375)

P10: „An online card game, I cannot think of the name now, unfortunately that is Covid related. I played that passionately, almost addictively, and with a lot of effort I got out of the habit, because I realized how long I spend playing and that is all life time and that is not good for me. I do not play other games at all/. I am not interested in that." (RZM_PEU2, paragraph 37)

Further examples from the interviews to theme 4 which are not cited in the main body of the manuscript

P05: „I think that is good, but it does not seem so easy and for me it would be very difficult to take part in it. I am interested, but I find it very demanding. Um, at least as my condition is now. Very difficult. I would almost be a little overwhelmed with certain things." (RHF_PEU1, paragraph 375)

P11: „We did not grow up with that." (RHF_PEU3, paragraph 327)

P11: „Well, I am probably um overwhelmed here because the stuff does not interest me at all, like the electronic games. I do not watch TV or stuff like that either, so I do not feel attracted to the screen at all." (RHF_PEU3, paragraph 53)

P11: „It has never interested me because no one has explained to me how it works. And with time, interest has been lost." (RZM_PEU2, paragraph 35)

P15: „Whereas, I have to say, that is a bit/ um, it still tends to be addictive." (RHF_PEU4, paragraph 89)

P10: „The left online card game, I cannot think of the name now, unfortunately that is Corona related. I played that passionately, almost addictively, and with a lot of effort I got out of the habit, because I realized how long I spend time there and that's all life time and that is not good for me. I do not play other games at all/ I am not interested in that." (RZM_PEU2, paragraph 37)

---

5. Narratively realistic training that focuses on activities of daily living is desired to manage daily living as independently as possible.

P10: „It was very close to reality, so it was easy to empathize. That was my first, my first thought was, that I could relate to it." (RZM_PEU2, paragraph 238)

P05: „Um there must be more stability (..) I am an insecure walker or (.) sometimes a little dizzy and so on and I am here to improve that with a therapist. Learning to walk properly again or and that is the main goal, that I walk again, independently (.) like before." (RHF_PEU1, paragraph 220)

Further examples from the interviews to theme 5 which are not cited in the main body of the manuscript

---

P10: „It was very close to reality, so it was easy to empathize. That was my first, my first thought was, that I could relate to it." (RZM_PEU2, paragraph 238)

P07: „The most important thing for me is to regain the ability to walk and to maintain my balance. For the main part, I have succeeded, except for walking, where I am still a bit wobbly. But that is getting better. I hope in a week, when my therapy stops, that it's then that I can put the walker aside and all the other therapies like treadmill and bike, that's something for me, (inc.) and I hope the whole thing gets even better." (RZM_PEU2, paragraph 185)

P05: „Um there must be more stability (..) I am an insecure walker or (.) sometimes a little dizzy and so on and I am here to improve that with a therapist. Learning to walk properly again or and that is the main goal, that I walk again, independently (.) like before." (RHF_PEU1, paragraph 220)

P05: „If I want to pursue all my hobbies again in reality, it would not be possible. You would have to build a tennis hall with an indoor tennis game, but that is already not possible. But you could do that on such a video thing/" (RZM_PEU1, paragraph 167)

---

6. The game environment distracts older adults from the physical and mental effort during training.

P08: „But I still see a big advantage with computer assisted video games, you can introduce practically any level or any performance standard. I can do penalty shootouts today against Christiano Ronaldo and this is facilitative, to make the game more exciting or increasingly more exciting. Or I can play tennis against Dominic Thiem. It is all conceivable. " (RZM_PEU2, paragraph 164).

P15: „Then it becomes difficult to maintain one's motivation."

I: „Yes, then here/ (.) so, evenly, if so/ if the progress stops/"

P15: „Yeah, if you do not see the progress." (RHF_PEU4, paragraphs 561-563)

Further examples from the interviews to theme 6 which are not cited in the main body of the manuscript

---

P12: „Whether it is tennis or fishing or anything else, it does not matter. You are supposed to respond to people's needs a little bit." (RZM_PEU2, paragraph 121)

P13: „It is/ just that as a human being I also want to succeed." (RHF_PEU3, paragraph 1185).

Secondary end users

1. **From the secondary end users' perspectives, a functional and individualized meaningful game design is of great importance to older adults for increasing training motivation.**

S02: „I also have the impression, that with many patients it is important WHAT problems they really have in everyday life, and if you really aim the training at what is important to them, be it now that they have their balance just as we just heard, they need it more in everyday life or in the kitchen, at home, or with sports, or if they simply train their balance specifically for THAT, where they need it, they are also more motivated and simply (...) more involved in the therapy than just doing some exercises, where they do not know where they need them in everyday life. "(RZM_SEU1, paragraph 21)

S08: „Somehow transfer that into the game. Or then you picked things up, did squats or something like that/."

S01: (laughs)

S08: „And then you walk through the store because you want to take something out of the bottom shelf. Well, I think that calls for such a system." (RHF_SEU2, paragraphs 1082-1084)

Further examples from the interviews to theme 1 which are not cited in the main body of the manuscript

S07: „They practice things that are relevant to THEM and recognize the benefit after a certain number of training sessions." (RHF_SEU1, paragraph 240)

S06: „I often have the feeling that people find it a bit DIFFICULT at the beginning when it has the character of a VIDEOgame, when there is a bit of a lack of reference to everyday life" (RZM_SEU2, paragraph 55)

S01: „Well, the forest would certainly be nicer than a completely virtual one/ (.) like there in sports." (RHF_SEU2, paragraph 1028)

2. The fall protection device in the ExerCube is expected to provide older adults with a feeling of safety that will allow them to train at their individual performance limits.

S03: „Being able to try out everything in a SAFE setting that the patient would no longer have DARED to do: walking backwards, walking forwards, walking sideways, changing quickly and then throwing himself in. That was where I think a lot of TRUST can be re-established, if the limit is secured." (RZM_SEU1, paragraph 52)

S02: „And I still find it difficult, especially when it comes to balance training/"

I: „Hm (agrees)."

S02: „And you then/ I mean, the Dividat is great, it has a railing around it where patients can hold on to. But if they hold on then we will not have the effect we want."

I: „Hm (agrees)"

S02: „And there so/ (.) to find such a/ such a good balance between safety and effectiveness. So, effective balance, I would find that kind of difficult." (RHF_SEU2, paragraphs 507-511)

Further examples from the interviews to theme 2 which are not cited in the main body of the manuscript

S02: „Well, you could also turn it around. No advantage would be if you/. If the tasks are too difficult.“

I: „Hm (agrees).“

S02: „Or too easy.“

I: „Hm (agrees).“

S02: „And I do not have to do anything for it. It must be nice on this threshold, where I just barely achieve something, but/.“

I: „Hm (agrees). Yes.“

S02: „Where I can get into a bit of a flow." (RHF_SEU2, paragraphs 736-742)

3. Based on the therapists' experience, digital gamification in therapy leads to an increased motivation in older adults.

S01: „Our feedback from MANY people is just like, 'What? The therapy is already finished yet?' And now, no, they want to keep playing because they are going to crack the high score again, and the motivation is just there." (RZM_SEU1, paragraph 76)

S03: „The advantage is that tasks are solved playfully, adopting an external focus. And by doing so, you almost forget the time or the effort. That is also the effect that you otherwise (.) had with the ball back then, if you do something with the ball, you forget the time and effort, yes. You do a lot more with it than when you say: 'now you run five laps in the hall'. That's a huge difference, yes." (RHF_SEU1, paragraph 178)

Further examples from the interviews to theme 3 which are not cited in the main body of the manuscript

S02: „Because it gives the movement a certain lightness, a playfulness." (RHF_SEU2, paragraph 214)

S03: „Yes, because the [brain’s] reward system, which is very important with stories like this, you have to stimulate it somehow, yes. Then there are two options. We have them, or you have (points to T04) developed the certificates/. Yes? I have one hanging in the room (T04 amazed, smiles, T06 smiles, I01 laughs), I think that's great! Yes. So you really just reward the performance that someone does, whether it is a training device, an exergame or something else. And you get a kind of reward there in the system, yes. A placement or funny hints or something. You can integrate all of that, depending on how your clientele is." (RHF_SEU1, paragraph 98)

S07: „He said: ‘ok, now I'm seeing it for the THIRD time’ and now he can perhaps ORIENTATE himself there in the world and then find what he DID NOT find the last time or something like that, then the sense of achievement is also there.“ (RZM_SEU2, paragraph 282)

4. The game environment distracts older adults from their functional limitations and allows them to unconsciously move more freely.

S01: „Well, for example, he is someone who really likes to walk on his heels. And now, when you bring the bees to the front/ (.) like, then you notice: Hopp, he is in trouble right away. But at least he does it. And sometimes during the exercises, no matter how I tell him: ‘Hey, now do not shift the weight forward with your forefoot’/ (.) difficult! (laughs)“

I: (laughs)

S01: „But here in this game, that is what happens." (RHF_SEU2, paragraphs 102-104)

Further examples from the interviews to theme 4 which are not cited in the main body of the manuscript

S09: „We were playing tennis and all of a sudden and then he got and that was like the Wii, we borrowed it and have it with him, he's already at the home himself and we were playing with the Wii back then and that was //.“<br>S10: „Yeah, Great.”<br>S09: „Suddenly he stands up, leaves the rollator where it is and moves FREELY. Of course, we already made sure that he SECURES himself somewhere, but we were never there, funnily enough we were never afraid that he was in any danger of falling.“ (RZM_SEU2, paragraphs 77 - 79)
S04: „when you suddenly find yourself standing in the kitchen. That would be great, of course, and it is a huge opportunity. That one forgets the rehab setting and thinks one is at home.” (RHF_SEU1, paragraph 42)
S06: „Yes, it is actually a great thing when you think to yourself, you are playing a game and you do not even notice that you are training with it, you know? That is actually what would be nice (smiles)." (RHF_SEU1, paragraph 97)
**5. The availability and perceived time demands of an exergame may limit its usability.**
S07: „For me it's also very important, or (smiles) almost a top priority, that the device works (collective laughter) properly. That may sound banal (S04 laughs), but it happens more often than you think. And then you just stand there with the patient and (.) there you are again with the attitude of the patient. If, as a therapist, you somehow have no idea what the problem is, then it affects the patient, and they might not like to train as much next time. So, it just has to work (.) or to be solved quickly (common laughter). (..) I see that as a bit of a barrier, that there are always technical errors, software things or maybe hardware issues.” (RHF_SEU1, paragraph 163)
S10: „Well, I think it definitely adds value if you (inc.) use the device, I think that is very exciting. Difficult setting everything up and adjust it individually to the patient but at the same time still be very easy to use, so that it is almost always works at the push of a button, that is the way it is, that would of course be the (laughter from everyone) ultimate highlight for a therapist." (RZM_SEU2, paragraph 393)
**Further examples from the interviews to theme 5 which are not cited in the main body of the manuscript**
S02: „and you only have (.) 25 minutes / then you do not want to make a fool of yourself or lose too much of the therapy time." (RHF_SEU2, paragraph 145)<br><br>S03: „If it is really a casual and very effective piece of equipment, then you just have a bit of a problem getting it when you need it." (RZM_SEU1, paragraph 133)<br><br>S06: „And if you still need any belts or anything that you can really (.) put it on promptly and do not have such a huge gadget with a thousand buckles to adjust it (S07 agrees) or something like that. That you really (.) get in effectively (.) quickly.” (RHF_SEU1, paragraph 165)
**6. A lack of local accessibility to and availability of the exergame after discharge from a rehabilitation center and an unawareness of alternative, non-computerized training strategies may influence adherence negatively.**
S08: „Yes, for the patients in inpatient settings it is always important that they can perhaps continue somewhere at home or in therapy. Well, you can see that/ I mean, if you/ (inc.) have equipment, then they always ask: ‘Ah, which private practice has it, too?’” (RHF_SEU2, paragraph 1142)<br><br>S02: „I think, in principle it is good as a SUPPLEMENT to therapy, I would definitely NOT REPLACE therapy with it, so that you only do the exergame now, um, the only danger I might see with something like that is that a certain dependency might occur, in the sense that not everyone has an exergame at home, which means

that you might use it in therapy or rehab in between, and if the patients really like it, they don't know how they can continue their training with it at home, whether it's coordination or whatever, um, and that they only want to play the exergame every time they come for therapy, but, It would be good if the patients also knew what they could do by THEMSELVES without having to rely on certain things, such as exergames." (RZM_SEU1, paragraph 88)

Further examples from the interviews to theme 6 which are not cited in the main body of the manuscript

S03: „That you just have a bit of a problem getting it whenever you need it." (RZM_SEU1, paragraph 133)

Tertiary end users

1. From the tertiary end users' perspectives, an evidence-based additional benefit is a prerequisite for financially supporting a research and development project, as well as for considering a financial reimbursement of exergame applications within rehabilitation.

T03: „If we talk about health insurances, we have the case, that they prefer to see evidence-based, that a real benefit is gained from using that device" (RZM_TEU3, paragraph 22)

T01: „On the one hand we obviously have the commission to achieve the optimum for our clients, for your patient, who is the same person, to be able to support the optimal possibility for healing. The financial component, however, is still the first priority for us, where there um, has to be some kind of, um (being appealing?) for it to become obvious: 'Okay, that is financially still attractive'. Maybe you could therefore also state, yes, maybe that could result in a reduction of the duration of being inpatient or something similar. It is not about inventing incentives in whatever way, but there should be a progress somehow/. There clearly has to be a progress, a considerable advantage derived from the training, which you could state. Therefore, we need some information, but for, for the support of pilot projects we are PRINCIPALLY open." (RHF_TEU1, paragraph 99)

Further examples from the interviews to theme 1 which are not cited in the main body of the manuscript

T02: „Um, ultimately the issue in that case is the user, right, of these, these exergames. (.) And these exergames are of course (.) / are deployed within an rehabilitation program, right, so there is already a package existing, right? And if that results only in higher costs of that package, then there is no chance, right? Therefore, it is really necessary to show benefit (.) MEASUREMENT or first proof the beneficial evidence, right? That there IS indeed an advantage producible of the application of this/this as of now specific PRODUCT." (RHF_TEU2, paragraph 40)

2. Tertiary end users desire diverse training applications and settings of the ExerG in order to reach heterogeneous target populations with different impairments and at various rehabilitation stages.

T03: „Um and for whom would that be suitable? If there is a certain case like patient A for an individual application and then there is another individual application for patient B and in the end it is only poorly and rarely in use., I think in that case it makes sense to say: Okay, you can apply it um reasonably throughout one's therapeutic treatment at various, I would call them levels of progression or stages of progression." (RHF_TEU3, paragraph 273)

T02: „And I think it is certainly EXCITING. (.)As I said before, you specifically have to select and choose, what people are we talking about if we talk about patients (.) right? Which indicators are given? (.) and therefore you cannot speak for ALL, right? But you have to select very specifically what patients are in need and could get something out of it." (RHF_TEU2, paragraph 32)

**Further examples from the interviews to theme 2 which are not cited in the main body of the manuscript**
T02: „Propagation will develop over various application fields, also into the direction of nursing homes, obviously“ (RZM_TEU2, paragraph 37)
**3. The main goal of an ExerG training should be older adults' return to an autonomous everyday life.**
T01: „Well, what I like about it, is that it is not based on, not machinery controlled, but for real you have to stand and walk, bend, like natural movements, needed in everyday life. And that is also presented in this way by those videos or these games. And that you regain your capacity of how to do the groceries and/ so far, I have not seen exactly, what / Or, when that person was doing the groceries, it will probably be presented to the player what you should // (buy?).” (RHF_TEU1, paragraph 191)<br><br>T02: „It is pleasurable, that the world presented is not a VIRTUAL one, in aspects of being presented as a game world, but being presented as a day-to-day-world, right? For example, being at (the river?) um, or in a supermarket, um, I think it is basically a good concept, (.) which may also simplify the access for people who are not that much into gaming, in my opinion” (RHF_TEU2, paragraph 92)
**Further examples from the interviews to theme 3 which are not cited in the main body of the manuscript**
T05: „And therefore it is our interest that, when I say, I am a baker and have to do these procedures permanently, slide those baking trays in and I have to ask for all these things within the occupational history, what do you need in relation to your occupation, in order to regain your capacity.” (RZM_TEU5, paragraph 20)<br><br>T05: „As I said before, these daily tasks with the supermarket, is something we already have and apply with the „Lokomat“, as well as within these gamification tools we used in [location has been removed due to data protection principles], where you arrange different things on a shelf and are happy about it. You have to take the trolley and steer it – for me, these are the things which are the most purposeful.” (RZM_TEU5, paragraph 38)
**4. Tertiary end users assume that older adults are reluctant to use exergames, due to a lack of experience in technology and therefore express themselves partially skeptical, critical and/or reserved about the use of the ExerG in rehabilitation settings.**
T05: „The first videos [original ExerCube exergame] were appropriate for young people, not for the older generation. This is not suitable for an old person, also rather dissuasive, if there are so many things which impact on you while standing in a virtual room and there are just too many different impressions. An older person is not capable of processing, and may feel more threatened rather than comfortable, without getting a real benefit out of it.” (RZM_TEU5, paragraph 36)<br><br>T02: „That person seems to be very lonely and lost in that Cube which could be less comfortable for older people, being surrounded by a virtual room and being alone there.” (RZM_TEU2, paragraph 52)
**Further examples from the interviews to theme 4 which are not cited in the main body of the manuscript**
T01: „Yes. (inc.) // patients, who are reluctant, but that is // maybe the challenge /.“<br><br>I: „Yes. //“<br><br>T01: „To also get those a little bit in the direction of these games, maybe they develop fun while playing (laughing). But probably there are patients who cannot get to that point. But those being a little bit more open-minded, I believe in the additional value of this intervention.” (RHF_TEU1, paragraphs 67-69)

5. A professional training of the therapist and patient-oriented training support during exergaming are considered vital for therapeutic success.

T01: „In the future, the personal contact within a therapeutic process must not be replaced completely, there should develop a combination with being trained or treated by another human being and I see it as an add-on instead of a replacement.” (RZM_TEU1, paragraph 27)

T03: „I am concerned that, let us say, okay, for example you play a game, where you perform squats (.), I would call it, make somebody jump. It does not matter, you perform whatever action. But then it is also important, that you check that: 'Like okay, (.) is that task completed correctly?' As you may get into the flow of gaming, not being able to follow the tasks appropriately and you make mistakes by performing your exercises, and in the end, you worsen more than you improve.” (RHF_TEU3, paragraph 64)

Further examples from the interviews to theme 5 which are not cited in the main body of the manuscript

T02: „Of course it is necessary that therapists instruct the patients appropriately as well as target-group-oriented.” (RZM_TEU2, paragraph 43)
